# Linking Student Performance in Massachusetts Elementary Schools with the “Greenness” of School Surroundings Using Remote Sensing

**DOI:** 10.1371/journal.pone.0108548

**Published:** 2014-10-13

**Authors:** Chih-Da Wu, Eileen McNeely, J. G. Cedeño-Laurent, Wen-Chi Pan, Gary Adamkiewicz, Francesca Dominici, Shih-Chun Candice Lung, Huey-Jen Su, John D. Spengler

**Affiliations:** 1 Department of Forestry and Natural Resources, College of Agriculture, National Chiayi University, Chiayi, Taiwan; 2 Exposure, Epidemiology and Risk Program, Department of Environmental Health, Harvard School of Public Health, Boston, MA, United States of America; 3 Environmental Occupational Medicine and Epidemiology Program, Department of Environmental Health, Harvard School of Public Health, Boston, MA, United States of America; 4 Department of Epidemiology, Brown University, Providence, RI, United States of America; 5 Department of Biostatistics, Harvard School of Public Health, Boston, MA, United States of America; 6 Research Center for Environmental Changes, Academia Sinica, Taipei, Taiwan; 7 Department of Atmospheric Sciences, National Taiwan University, Taipei, Taiwan; 8 Department of Environmental and Occupational Health, College of Medicine, National Cheng Kung University, Tainan, Taiwan; Tilburg University, Netherlands

## Abstract

Various studies have reported the physical and mental health benefits from exposure to “green” neighborhoods, such as proximity to neighborhoods with trees and vegetation. However, no studies have explicitly assessed the association between exposure to “green” surroundings and cognitive function in terms of student academic performance. This study investigated the association between the “greenness” of the area surrounding a Massachusetts public elementary school and the academic achievement of the school’s student body based on standardized tests with an ecological setting. Researchers used the composite school-based performance scores generated by the Massachusetts Comprehensive Assessment System (MCAS) to measure the percentage of 3^rd^-grade students (the first year of standardized testing for 8–9 years-old children in public school), who scored “Above Proficient” (AP) in English and Mathematics tests (Note: Individual student scores are not publically available). The MCAS results are comparable year to year thanks to an equating process. Researchers included test results from 2006 through 2012 in 905 public schools and adjusted for differences between schools in the final analysis according to race, gender, English as a second language (proxy for ethnicity and language facility), parent income, student-teacher ratio, and school attendance. Surrounding greenness of each school was measured using satellite images converted into the Normalized Difference Vegetation Index (NDVI) in March, July and October of each year according to a 250-meter, 500-meter, 1,000-meter, and 2000-meter circular buffer around each school. Spatial Generalized Linear Mixed Models (GLMMs) estimated the impacts of surrounding greenness on school-based performance. Overall the study results supported a relationship between the “greenness” of the school area and the school-wide academic performance. Interestingly, the results showed a consistently positive significant association between the greenness of the school in the Spring (when most Massachusetts students take the MCAS tests) and school-wide performance on both English and Math tests, even after adjustment for socio-economic factors and urban residency.

## Introduction

The relationships among human health and well-being, biodiversity, healthy ecosystems, and a changing climate have received increasing attention in recent years in international discussions and policy processes [1]. The Millennium Ecosystem Assessment, for example, compiled a list of ecosystem goods and services that are crucial for human survival [2], such as forests that produce oxygen, store carbon dioxide, and restore degraded ecosystems [1], [3]. Although the underlying pathways of the effects of green spaces on health are not fully understood, a number of benefits may result, such as increased physical activity, increased social contacts, reduced psychophysiological stress and depression, decreased noise, microclimate regulation (moderation of ambient temperature and urban heat island effects), and reduced air pollution levels [1], [4]–[13]. These benefits from exposure to green spaces can be expected to translate into a supportive environment for academic achievement in children.

For many years, academic researchers have tried to identify determinants of student performance and analyze which variables impact student performance positively and negatively [14]–[17]. Gender, ethnicity, and father’s occupation have been identified as significant contributors [18]; Chambers and Schreiber identified a gap between the achievement of boys and girls, with girls showing better performance than boys under certain instances [19]. Above and beyond the other demographic factors, socio-economic status shows a significant effect at the individual level [20]. While socio-economic status may be defined in a number of different ways, it usually entails a combination of parental education, occupation, income, and facilities used by individuals separately or collectively. Students with high socio-economic status perform better than middle-class students, and middle-class students perform better than the students at the lower end of the socio-economic scale [16], [21]–[23]. In addition, economically disadvantaged parents are less able to afford the cost of their children’s higher education [24]–[25]. No research, however, has examined the specific effects of green surroundings on academic performance.

**Figure 1:**
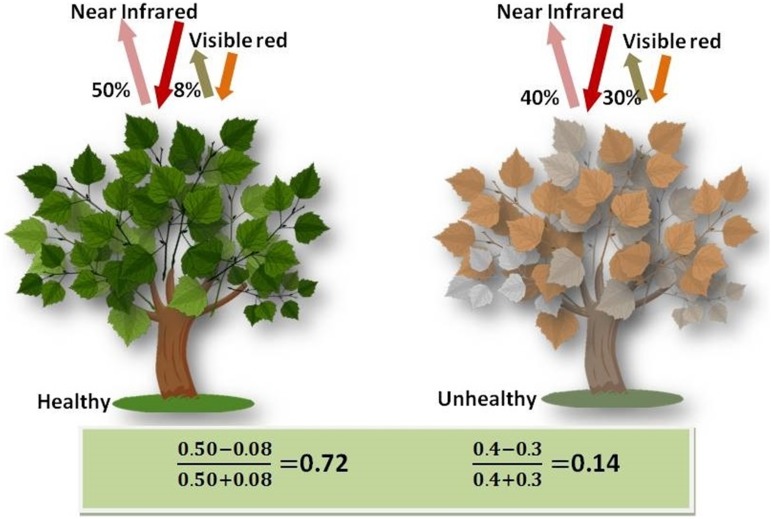
Green vegetation (left) absorbs visible light and reflects near-infrared light; Sparse vegetation (right) reflects more visible light and less near-infrared light. The NDVI is the ratio of absorbed visible light and reflected near-infrared to the total amount of visible and near infrared radiation striking a surface.

Today’s readily available remote sensing technologies easily and effectively provide large-scale and multi-temporal surface information for many purposes, including a forest greenness assessment. The Normalized Difference Vegetation Index (NDVI) is a spectrum-based greenness index that measures and monitors plant growth (vigor), vegetation cover, and biomass production [26]. It is based on the reflected spectral responses from two different wavebands, one in the red (0.6–0.7 µm) and the other in the near-infrared (NIR) (0.7–1.1 µm), as shown in Fig. 1. Vigorously growing healthy vegetation (left) has low red-light reflectance and high near-infrared reflectance, and hence, high NDVI values. Sparse vegetation (right) reflects more visible light and less near-infrared light. A simple algorithm produces output values in the range of −1.0 to 1.0. Increasing positive NDVI values indicate increasing amounts of healthy green vegetation. NDVI values near zero and negative values indicate non-vegetative features, such as barren surfaces (rock and soil), water, snow, ice, and clouds.

In prior studies, NDVI (“greenness”) has been associated with a number of health outcomes. Neighborhood greenness has been associated with of adult weight [27], birth weights [28], cardiovascular disease [29], cumulative stress [30], mortality [31], and personal exposure to air pollution among pregnant women [4].

This is the first study to investigate the link between surrounding greenness and school-based performance scores using remote sensing techniques.

## Materials and Methods

### Study area

**Figure 2:**
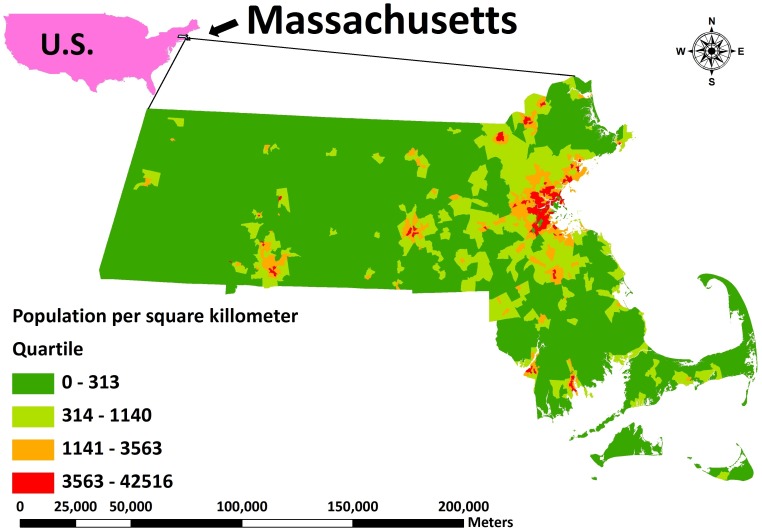
Massachusetts population distribution based on the 2010 U.S. Census. Red to green indicate higher to lower populated areas.

Massachusetts, located in the northeastern United States, is bordered by Rhode Island and Connecticut to the south, New York to the west, Vermont and New Hampshire to the north, and the Atlantic Ocean to the east. It is the 7^th^ smallest of the 50 states in landmass, but it is the 14^th^ most populous and the 3^rd^ most densely populated. Most of the state’s residents live in the Boston Metropolitan Area, also known as Greater Boston, which includes New England’s two largest cities, Boston and Worcester. With more than 100 universities in Massachusetts, including Harvard University and the Massachusetts Institute of Technology (MIT), Massachusetts is one of the world’s most important educational centers. Fig. 2 shows Massachusetts’ population distribution based on the 2010 U.S. Census.

### School level academic performance

Data from the Massachusetts Comprehensive Assessment System (MCAS) provided the school-based measure of student performance. Developed in 1993 by the Massachusetts Department of Education, MCAS provides a standard evaluation of student, school, and district performance. Its Composite Performance Index (CPI) is a measure of the extent to which students are progressing toward proficiency. (Refer to the Massachusetts Department of Elementary and Secondary Education, http://www.doe.mass.edu/mcas/for more information.) The CPI is the percentage of 3^rd^-grade students (8–9 years old) scoring “Above Proficient” (AP) in English and Math. AP is the highest level given to third graders. It means that students have learned challenging subject matter and can construct solutions to challenging problems [34]. In addition, the researchers collected socio-demographic data from the MCAS database, such as race, gender, English as a second language (proxy for ethnicity and language facility), family income level, student-teacher ratio, and school attendance.

Since recent studies have indicated that the proximity to water can improve physical and mental health [32]–[33], this study excluded schools surrounded by water, such as, records in the database with negative values of vegetation index (n = 256, about 37 schools per year, or less than 5% of the records) to avoid misclassification bias. In total, this study analyzed school-level CPI data from 905 Massachusetts public schools collected between 2006 and 2012 (n = 6333). Moreover, home greenness could be an important factor to students’ performance. Due to the privacy issue, the Massachusetts Department of Education never releases personal information for individual student. Estimation of home greenness becomes problematic because of the lack of students’ home addresses. To deal with this issue, only public schools were included in our analysis. Since students under the public system of state education are required to attend the most adjacent school in the school district, home greenness exposure was also considered in the buffer analysis from the schools.

### GIS data of schools

Determining the green buffer surrounding the schools required a GIS data layer of schools obtained from MassGIS (http://www.mass.gov/mgis) that shows the central coordinates of schools in Massachusetts serving students from pre-kindergarten through high school.

### Green exposure

The amount of trees and vegetation (“greenness”) in the vicinity of schools was obtained from NASA’s Earth Observing System data – the global Moderate Resolution Imaging Spectroradiometer (MODIS) NDVI. This system calculates the global distribution of vegetation types, as well as their biophysical and structural properties and spatial/temporal variations. NASA provides Global NDVI data updates every 16 days at 250-meter spatial resolution as a gridded product in the sinusoidal projection [35]. Since NDVI Version-5 products are validated Stage 2, meaning that accuracy has been assessed over a widely distributed set of locations and time periods via several ground-truth and validation efforts, they are ready for use in scientific publications [36].

**Figure 3:**
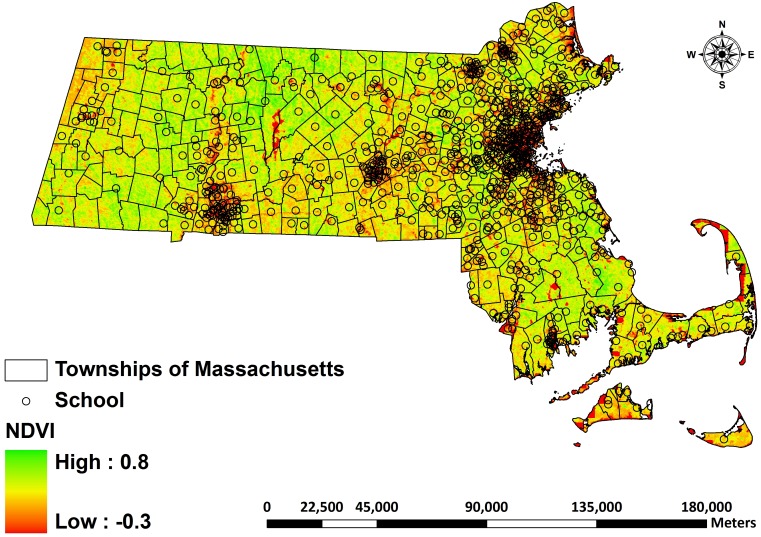
NDVI values for year 2012 (March 21) and location of schools included in this study. Green to red represents the greenness level from high to low.

This study used NDVI data from March (March 21 or 22) and October (October 15 or 16) of each study year to quantify greenness exposure in the spring and fall of the school year. The study also collected NDVI readings from July (July 27 or 28) because that period would show the maximum “greenness” exposure, such as the most amount of vegetation growth available in that area [37]. The researchers abstracted surrounding greenness from 250-meter, 500-meter, 1000-meter, and 2000-meter circular buffers for each school to represent student’s exposure to greenness at school. Fig. 3 illustrates the spatial variability of NDVI for Massachusetts based on the NDVI map of March 21, 2012, and it shows the locations of schools included in this study. Please check File S1 for the details of the buffer analysis for greenness exposure assessment (Fig. A in File S1). Table A in File S1 is the summary of data used in this study.

### Statistical analysis

We hypothesized that the school’s proximity to greenness has a positive effect on student performance. Spatial Generalized Linear Mixed Models (GLMMs) were used to estimate the impacts of surrounding greenness on school performance. GLMMs are generalized linear models (GLMs) in which the linear predictor may contain random effects and within-group errors may be spatially autocorrelated [38]. In cases where spatial data are available from several disjunct regions, GLMMs can be used to fit overall fixed effects while spatial correlation structures are nested within regions, allowing the accommodation of regional differences in e.g. autocorrelation distances, and assuming autocorrelation only between observations within the same region [39]–[42]. In this study, the researchers considered Spatial Autocorrelation (SAC) given the high densities of schools in very similar areas. To deal with this issue, an additional term was added in the GLMMs for “county” to account for schools within a similar geographical region [43]. The global Moran’s I was calculated to test the significance of SAC in our models, and the results are shown in Table B in File S1. We did not find influential SAC in the models that incorporated the term of county of the schools.

This study adopted the percentages of AP students in English and Math respectively as the independent variables for representing the performance of the schools. As for the surrounding greenness, the range of NDVI data used in our analysis ranged from zero to positive one since schools with negative NDVI values were excluded. Adjustments were made using representations of a range of well-established factors [44] obtained from the MCAS, including race (percentage of different populations at a school, including African American, Asian, Hispanic, White, Native American, Native Hawaiian, and Non-Hispanic), gender (percentage of female student), English as a second language (proxy for both ethnicity and language facility), family income level (percentage of students from low income families), student/teacher ratio, school attendance, and spatial location (county of schools). P-value of <0.05 was considered for statistical significance. R statistical package was used for all the analysis.

### Sensitivity analysis

We used three approaches to confirm the robustness of the model’s estimate for greenness effects. The first approach developed models by incorporating NDVI from three periods at various buffer distances. Coefficients of NDVI from these models were then compared to understand the relationship between spatial-temporal greenness exposure and overall student academic performance by school. The second approach used only schools in Boston, the capital city of Massachusetts, and schools in Massachusetts’ five major cities – Boston, Worcester, Springfield, Lowell, and Cambridge – to develop the GLMMs for understanding the effects of study population (urban, suburban or rural) on model estimations, respectively (Table 1). The third approach duplicated the same analyses by using the averaged CPI score of schools instead of the percentage of AP students to test the robustness of the observed association to the definition of the outcome.

**Table 1:** Population of the five major cities of Massachusetts in 2013.

City	Population (%)
Boston	645,966(9.72%)
Worcester	182,544(2.75%)
Springfield	153,703(2.31%)
Lowell	108,861(1.64%)
Cambridge	107,289(1.61%)
Total	1,198,363(18.03%)

The number in parenthesis indicates the percentage relative to the total population of Massachusetts (information obtained from US Census Bureau 2013).

### Stratified analyses

This study used family income level and gender as the stratification variables [16], [19], [21], stratifying the entirety of the data into two groups by the median of the two variables, respectively, such as poorer/richer schools, and more females/fewer females schools. The results were then applied to assess the effect of these different strata of schools on greenness effects.

## Results

Table 2 shows the descriptive statistics of the study schools. In the past seven years, students in Massachusetts performed slightly better in English than in Math. About 35% and 16% of students were low-income and non-native English speakers, respectively. There were more boys at the schools. The student/teacher ratio indicates that the number of students was one order of magnitude larger than that of teachers. The averaged attendance rate of 3^rd^ grade students in Massachusetts were consistently high over the study period. Finally, White and Hispanic were the two largest student populations.

**Table 2:** Descriptive statistics of the 905 schools in Massachusetts during 2006 to 2012 (n = 6333).

Category	Variable	Mean ± Standard Deviation	Range
School performance	% Above proficient students of English	59.54±19.35	47.00 to 100.00
	% Above proficient students of Math	60.76±19.65	48.00 to 100.00
Known factors	% Low-Income	34.57±29.68	8.60 to 99.20
	% First Language Not English	16.28±19.15	2.10 to 93.80
	% Females	48.41±3.09	46.50 to 63.50
	Student/Teacher Ratio	14.11±2.61	12.50 to 72.90
	% Attendance	95.48±1.34	94.90 to 100.00
Race//Ethnicity	% African American	8.35±13.38	1.10 to 88.7
	% Asian	5.56±7.77	1.20 to 70.80
	% Hispanic	15.98±21.07	2.50 to 98.90
	% White	67.10±29.52	48.05 to 100.00
	% Native American	0.26±0.48	0 to 7.10
	% Native Hawaiian	0.14±1.02	0 to 67.90

Table 3 shows the GLMMs for the composite English and Math scores of the 3^rd^ grade students in the school. After adjustments for the available socio-economic factors, surrounding greenness in March showed a very significant (p<0.01) association with school academic achievement in English and Math regardless of which buffer distance was considered. Since most schools administer the MCAS tests in March/April, greenness in March describes the most contemporaneous relationship with academic performance, and therefore, this measure is particularly important.

**Table 3:** Coefficients (estimates with 95% confidence interval) of NDVI of (A) March, (B) July, and (C) October in GLMMs.

(A)			
March			
English		Math	
NDVI Buffer	Coefficient	NDVI Buffer	Coefficient
250 m	0.19 (0.16, 0.21)**	250 m	0.20 (0.16, 0.23)**
500 m	0.30 (0.27, 0.34)**	500 m	0.24 (0.19, 0.28)**
1000 m	0.38 (0.34, 0.42)**	1000 m	0.30 (0.26, 0.35)**
2000 m	0.42 (0.38, 0.46)**	2000 m	0.32 (0.27, 0.37)**
**(B)**			
**July**			
**English**		**Math**	
**NDVI Buffer**	**Coefficient**	**NDVI Buffer**	**Coefficient**
250 m	0.06 (0.04, 0.08)**	250 m	0.09 (0.07, 0.12)**
500 m	−0.001 (−0.03, −0.02)	500 m	0.05 (0.02, 0.09)**
1000 m	0.02 (−0.02, 0.05)	1000 m	0.06 (0.02, 0.10)**
2000 m	0.04 (0.01, 0.08)**	2000 m	0.05 (0, 0.09)*
**(C)**			
**October**			
**English**		**Math**	
**NDVI Buffer**	**Coefficient**	**NDVI Buffer**	**Coefficient**
250 m	0 (−0.02, 0.02)	250 m	−0.01 (−0.03, 0.02)
500 m	−0.06 (−0.08,−0.03)**	500 m	−0.04 (−0.07,−0.01)*
1000 m	−0.12 (−0.15,−0.09)**	1000 m	−0.07 (−0.10,−0.03)**
2000 m	−0.17 (−0.21,−0.14)**	2000 m	−0.11 (−0.15,−0.07)**

The coefficients are adjusted for race (percentage of different populations at a school, including African American, Asian, Hispanic, White, Native American, Native Hawaiian, and Non-Hispanic), gender (percentage of female student), language ability (percentage of first language not English), income level (percentage of low income student), student/teacher ratio, attendance, and location (county of school) in generalized linear mixed models.

*indicates P-value<0.05;

**indicates P-value<0.01.

Using the July and October NDVI measures in the analysis, the positive values of the parameter estimates for NDVI indicate that students with higher exposure to greenness for the balance of the year (even in summer) show better academic performance too with most of the estimates showing statistically significant results (p<0.05). Notably, many of the values in the month of October were negative but approaching zero (the absolute values of estimate were from −0.17 to 0) suggesting that the effects of surrounding greenness in October might not be clear.

Further evaluation of the impacts of buffer sizes on the parameter estimates of NDVI showed that NDVI in March, while statistically significant in all of the models for both English [E] and Math [M] (p<0.01), also showed an upward trend with greater NDVI coefficients at larger buffer distances: 250 meters (0.185[E] and 0.196[M]); 500 meters (0.3[E] and 0.236[M]; 1,000 meters (0.38[E] and 0.304[M]); 2,000-meters, (0.417[E] and 0.321[M]). Similar results were obtained from the models for Boston and the five majors cities (Table C in File S1). That is, the positive association between greenness and school performance remained significant in both English and Math while different buffer distances were considered and also the estimates of NDVI increased with larger buffer distances. The results of these sensitivity analyses that included different buffer zones and an evaluation of urban schools only were generally consistent with our main findings and supported the association between surrounding greenness and school-based academic performance.

**Figure 4:**
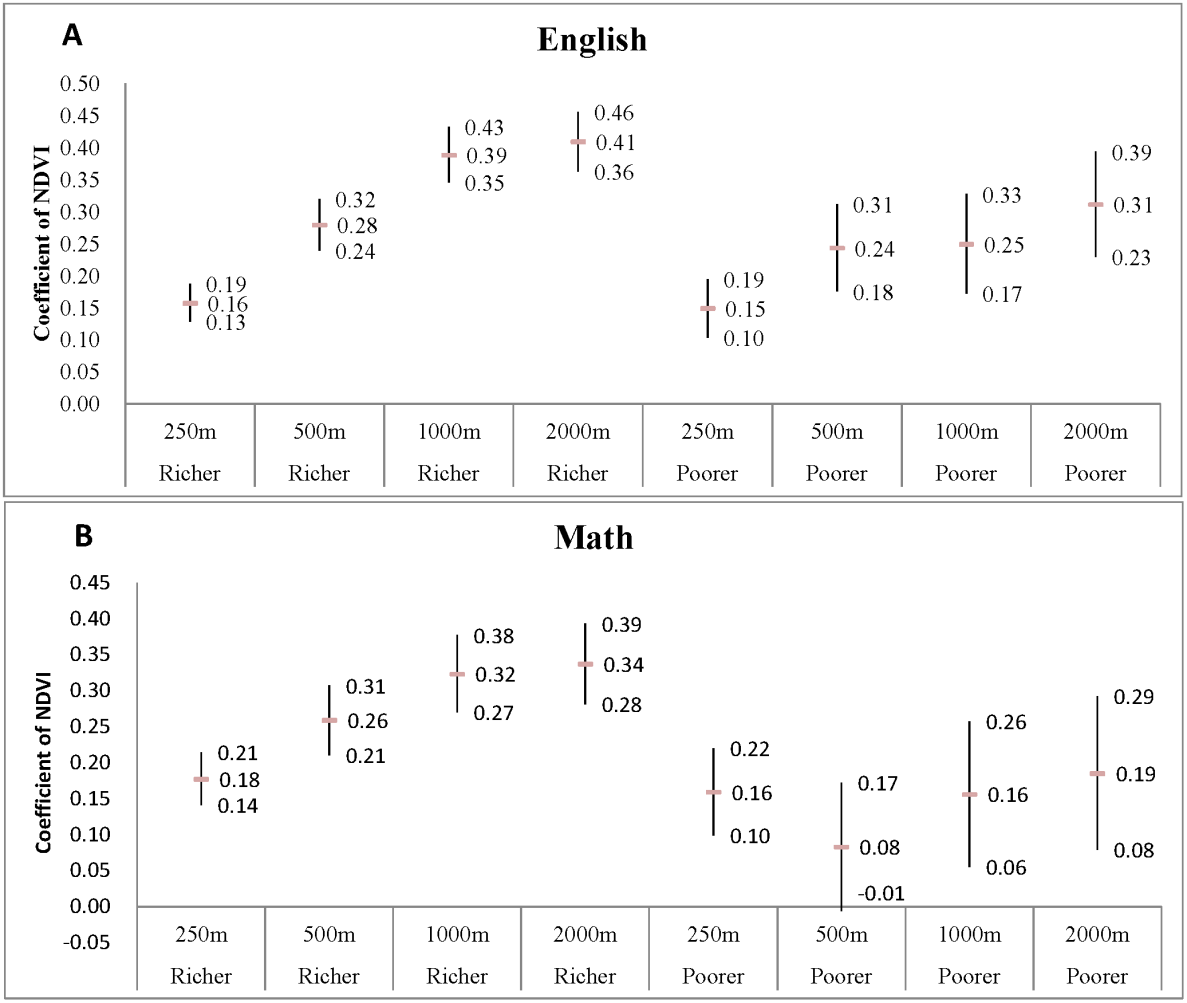
Stratified analysis for NDVI coefficients (estimates with 95% confidence interval) for (A) English and (B) Math models according to the median of percentage of low income of the study schools. The categorized female variable is based on the median levels of low income percentage (above or below median). NDVI is highly significant in all of the models (p<0.01).

**Figure 5:**
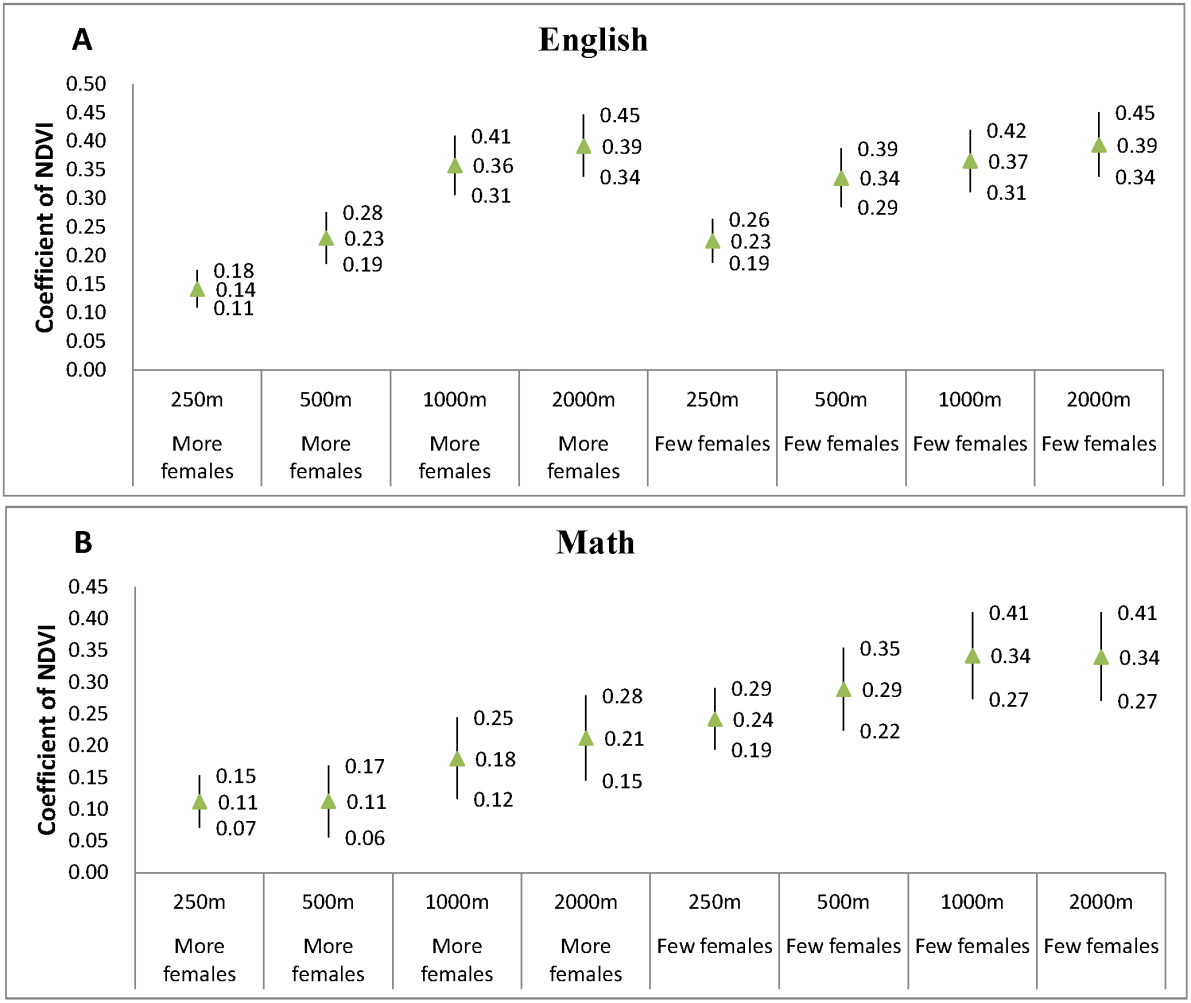
Stratified analysis for NDVI coefficients (estimates with 95% confidence interval) for (A) English and (B) Math models according to the median of percentage of female of the study schools. The categorized female variable is based on the median levels of female percentage (above or below median). NDVI is highly significant in all of the models (p<0.01) excepted for the estimate for poorer/math with 500 m buffer distance (p = 0.07).

Fig. 4 and Fig. 5 show the results of the stratified analyses based on the two stratification variables: family income level and gender. Basically, only minor differences on the NDVI estimates were found between different strata, suggesting that surrounding greenness has approximately equal effects on student academic performance regardless of financial status or gender.

Table D in File S1 shows the results based on the CPI scores. Generally, consistent results were obtained with our main findings, that is, surrounding greenness of March showed a very significant (*p*<0.01) association with student academic achievements in the two relevant subjects regardless of which buffer distance was considered. The estimates of NDVI of March was increased as a larger buffer was adopted. The results support that the observed association is robust to the definition of outcome of students’ performance.

## Discussion

This study used the public school performance database obtained from MCAS to examine the association between surrounding greenness and academic performance. MCAS provides a long-term and well-developed school/school district-level database that includes information of average standardized test scores and socio-economic variables from public schools across Massachusetts. We obtained the location coordinates for the schools from the official GIS database and the greenness area around the schools was defined according to various circular buffer distances from the center of the school.

Results from the model estimates of GLMMs and the sensitivity analyses were that surrounding greenness in March showed a significant (p<0.01) association with school-based academic achievement in both English and Math regardless of which buffer distance was considered. Moreover, the parameter estimate of NDVI was increasing as a larger buffer was adopted. Since students of public schools are usually assigned to schools near their homes, a buffer distance such as 2000-m may more accurately reflect the full exposure to greenness that is part of the school neighborhood where children likely spend their time when not in school. The logical conclusion is that greater estimates in the association between greenness and performance with increased size of the buffering zones in the NDVI measures comes from a more complete capture of the exposure to greenness.

It is interesting that our model estimates in October indicates opposite health effects of greenness from those of March and July. One of the potential reasons to explain the negative correlations between NDVI and student performance in October is because of the “Fall effect” in New England. Specifically, since the NDVI effectively measures the chlorophyll content of the leaves (photosynthetic capacity), otherwise green deciduous trees will appear “dead”–as the leaves will be falling off their maturity. Paradoxically, “greener school areas” will have higher red values in the Fall because of the changing color and dying of the leaves. Outside of the Fall and Winter season, such as in the Spring (March) or Summer, one would expect deciduous trees to have a low red-light reflectance and high near-infrared reflectance, and hence, high NDVI values. In the Fall, these trees will reflect more visible light and less near infrared light resulting in lower NDVI values. Thus, NDVI becomes a poor predictor of greenness in the Fall in New England. However, since the MCAST tests were took place in Spring, the model estimates of March strongly proved the positive effects of greenness on student performance.

Previous studies have determined that socio-economic factors such as family income, are important in influencing student achievement [18], [19]. Our findings, show that the association between NDVI and student performance proved to be statistically significant even after adjustment for income levels, gender, and levels of urbanization.

This study faced some relevant limitations. We did not have access to the raw individual student scores nor to individual student behavior, such as study time per day or time dedicated to family responsibilities or outside activities. Moreover, a number of socioeconomic indicators that have been predictive of student performance were not available, such as parental education [14], [15]. We did however obtain information about parental income in our models and this information may be sufficient considering that educational attainment has been shown to predict weekly earnings. For example, Table E in File S1, shows earnings by educational attainment released by U.S. Bureau of Labor Statistics [45]. A clear association could be observed between education level and median weekly earnings. This indicates that income level could be an alternative to educational attainment.

Moreover, we are also aware of the limitation of this study that is the cross-sectional design in an ecological setting which may suffer the caveats of confounding bias due to ecological fallacy. In this study, we have adjusted for the potential factors which may affect the students’ performance in the regression model to minimize the potential bias. The major findings are consistent with those in the sensitivity and stratified analyses. Although the findings in the aggregated level (school) may not be generalized to the individual levels (children), however, it is still a robust finding suggesting the effect of greenness on children’s school performance that could be informative for the public policy making.

As with the other studies that applied a remotely sensed methodology to measure the surrounding greenness [4], indoor greenness could not be accounted for by using the satellite based NDVI index, so indoor greenness could not be addressed in this analysis. Acquiring ancillary information about indoor greenness or developing a methodology for estimating indoor greenness must be an important issue for future greenness exposure assessments.

Finally, Massachusetts’ public schools may not be representative of schools in other states or countries in terms of outdoor climate and vegetation or indoor settings, school rules or facilities, including adjacent school playgrounds.

## Conclusion

This study applied remote sensing techniques and long-term student performance database to investigate the effects of surrounding greenness on the academic performance of the 3^rd^ grade students in Massachusetts. The results showed that students with higher exposure to greenness show better academic performance in both English and Math. More research along these lines is needed in additional locations and with more extensive academic performance data among various grades to determine the effects of greenness under different education systems. We suggest that future studies could duplicate the same analyses in different countries with various grades of students to further assess the effects of geographical differences and education systems on the benefits of green spaces. Moreover, we also recommend a cohort study design once the individual exposure data and performance records become available.

## Supporting Information

File S1 Supplemental Information. **Figure A,** Buffer zones from the central point of schools. **Table A,** Summary of data used in this study. **Table B,** P-value for Moran’s I from GLMMs models. **Table C,** Coefficients of NDVI of the models for schools in (a) Boston and (b) five major cities (Boston, Worcester, Springfield, Lowell, and Cambridge). **Table D,** Earnings by educational attainment of USA. **Table E,** Coefficients of NDVI of (a) March, (b) July, and (c) October in GLMMsa by using the CPI scores as the depended variable.
(DOCX)
