# Supplementary material for: Linking Student Performance in Massachusetts Elementary Schools with the “Greenness” of School Surroundings Using Remote Sensing
Source: PLoS One. 2014 Oct 13;9(10):e108548. doi: 10.1371/journal.pone.0108548 (PMC4195655; doi:10.1371/journal.pone.0108548)
Supplement: File S1 — Supplemental Information. Figure A, Buffer zones from the central point of schools. Table A, Summary of data used in this study. Table B, P-value for Moran’s I from GLMMs models. Table C, Coefficients of NDVI of the models for schools in (a) Boston and (b) five major cities (Boston, Worcester, Springfield, Lowell, and Cambridge). Table D, Earnings by educational attainment of USA. Table E, Coefficients of NDVI of (a) March, (b) July, and (c) October in GLMMsa by using the CPI scores as the depended variable. (DOCX) [file pone.0108548.s001.docx]

**Linking Student Performance in Massachusetts Elementary Schools with the "Greenness" of School Surroundings using Remote Sensing**

Chih-Da Wu^1,2^, Eileen McNeely^3^, J.G. Cedeño-Laurent^2^, Wen-Chi Pan^4^, Gary Adamkiewicz^2^, Francesca Dominici^5^, Shih-Chun Candice Lung^6,7^, Huey-Jen Su^8,*^, and John D. Spengler^2^

^1^ Department of Forestry and Natural Resources, College of Agriculture, National Chiayi University, Chiayi, Taiwan.

^2^ Exposure, Epidemiology and Risk Program, Department of Environmental Health, Harvard School of Public Health, Boston, MA, USA.

^3^ Environmental Occupational Medicine and Epidemiology Program, Department of Environmental Health, Harvard School of Public Health, Boston, MA, USA.

^4^ Department of Epidemiology, Brown University, Providence, RI, USA.

^5^ Department of Biostatistics, Harvard School of Public Health, Boston, MA, USA.

^6^ Research Center for Environmental Changes, Academia Sinica, Taipei, Taiwan.

^7^ Department of Atmospheric Sciences, National Taiwan University, Taipei, Taiwan.

^8^ Department of Environmental and Occupational Health, College of Medicine, National Cheng Kung University, Tainan, Taiwan.

*Corresponding author:

Professor Huey-Jen, Su, Department of Environmental and Occupational Health,

College of Medicine, National Cheng Kung University, 138 Sheng-Li Rd, Tainan 704, Taiwan.

Tel.: +886-6-2752459; Fax: +886-6-2743748. E-mail address: hjsu@mail.ncku.edu.tw.

Number of pages: 12

Number of figures: 1

Number of tables: 5

**Buffer analysis for greenness exposure assessment**

The GIS layer of schools was obtained from the Office of Geographic Information (MassGIS), which is the official institute of Massachusetts government for GIS database management. Location of the central point of each school was recorded in this dataset. Four kind of circular buffer zones including 250-m, 500-m, 1000-m, and 2000-m, were generated based on the central coordinate of the schools. The averaged NDVI values were then calculated based on the grids located within the buffers for representing the greenness exposure at different buffer distances.


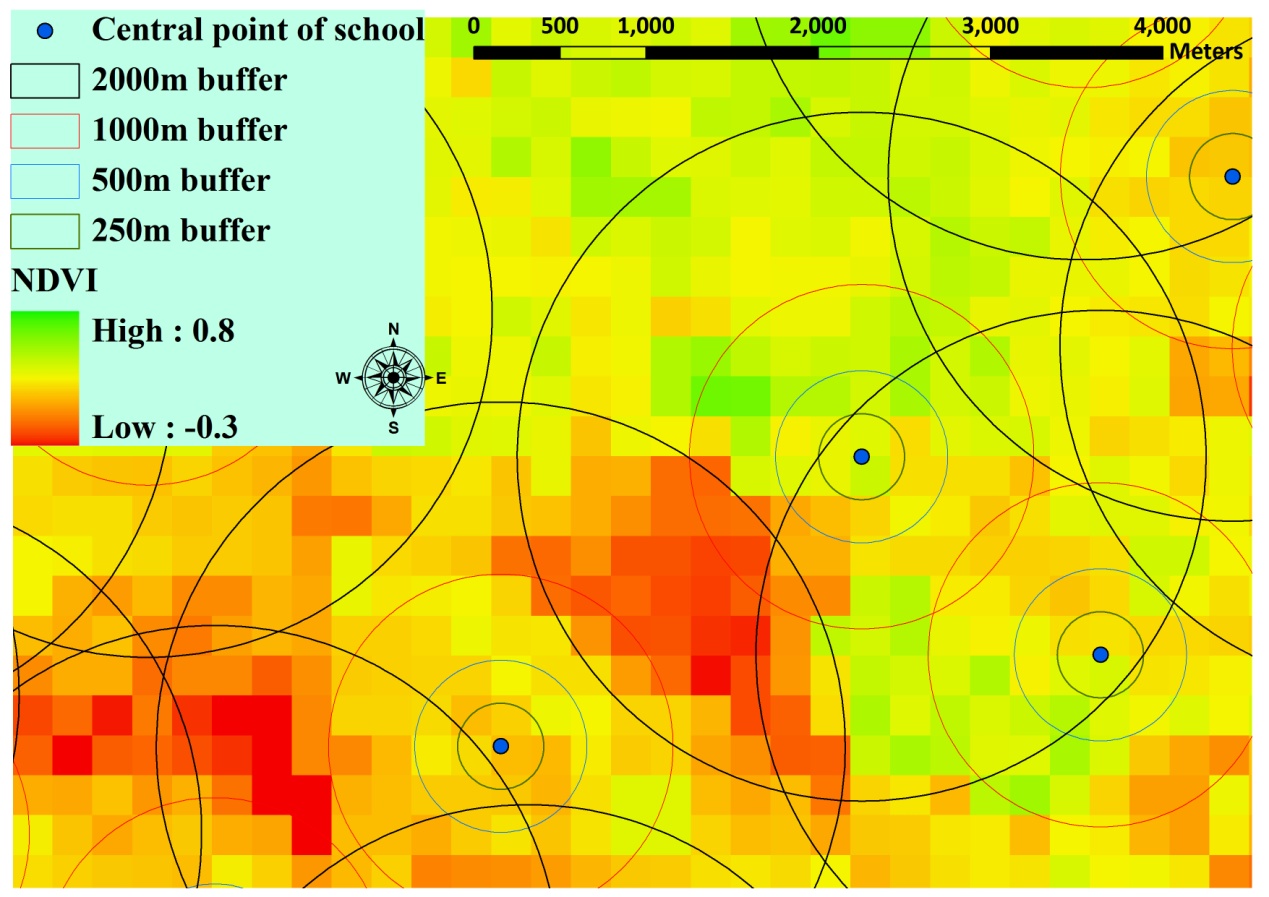


**Figure A. Buffer zones from the central point of schools.**

**The definition of the covariate of "Income level"**

The covariate of "income level" indicates the percentage of low-income students in the class. The following is the definition of low-income student from the Massachusetts Department of Elementary and Secondary Education (ESE) [1]:

**Low-income:** Indicates the percent of enrollment who meet ANY ONE of the following definitions of Low-income:

The student is eligible for free or reduced price lunch; or

The student receives Transitional Aid to Families benefits; or

The student is eligible for food stamps

**References**

1. Massachusetts Department of Elementary and Secondary Education (ESE). (2013) School/district profiles- Profiles Help - About the Data. Available: http://profiles.doe.mass.edu/help/data.aspx?section=students. Accessed 21 June 2014.

**Table A. Summary of data used in this study.**

| Database | Source | Description |
| --- | --- | --- |
| MODIS NDVI database (Ver. 5) | U. S. Geological Survey (USGS)  https://lpdaac.usgs.gov/ | NASA provides Global NDVI data updates every 16 days at 250-meter spatial resolution as a gridded product in the Sinusoidal projection . NDVI data from March (March 21 or 22), October (October 15 or 16), and July (July 27 or 28) were employed for the analyses |
| GIS data-   locations of schools | MassGIS  <http://www.mass.gov/mgis> | Location of school in Massachusetts and their attributes such as county of school are recorded in an Arcgis shapefile format. |
| Student performance | Massachusetts Comprehensive Assessment System (MCAS)  http://www.doe.mass.edu/ | Percentage of 3rd-grade students (8-9 years old) scoring "Above Proficient" (AP) in English and Math from 2006 to 2012 were used in this study. |
| Socio-demographic data | Massachusetts Comprehensive Assessment System (MCAS)  http://www.doe.mass.edu/ | Including race (percentage of different populations at a school, including African American, Asian, Hispanic, White, Native American, Native Hawaiian, and Non-Hispanic), gender (percentage of female student), language ability (percentage of first language not English), income level (percentage of low income student), student/teacher ratio, and attendance |

**Table B. P-value for Moran's I from GLMMs.**

| Year | Account for  Temporal | Account for  Spatial | Account for  Temporal and Spatial |
| --- | --- | --- | --- |
| 2006 | 0.02 | 0.53 | 0.63 |
| 2007 | 0 | 0.35 | 0.32 |
| 2008 | 0.31 | 0.33 | 0.76 |
| 2009 | 0 | 0.53 | 0.30 |
| 2010 | 0 | 0.00 | 0 |
| 2011 | 0.10 | 0.53 | 0.15 |
| 2012 | 0.41 | 0.40 | 0.69 |

**Table C. Coefficients (estimates with 95% confidence interval) of NDVI of the models for schools in (A) Boston and (B) five major cities (Boston, Worcester, Springfield, Lowell, and Cambridge).** The coefficients are adjusted for race (percentage of different populations at a school, including African American, Asian, Hispanic, White, Native American, Native Hawaiian, and Non-Hispanic), gender (percentage of female student), language ability (percentage of first language not English), income level (percentage of low income student), student/teacher ratio, attendance, and location (county of school) in generalized linear mixed models. ** indicates P-value < 0.01.

(A)

| English |  | Math |  |
| --- | --- | --- | --- |
| NDVI Buffer | Coefficient | NDVI Buffer | Coefficient |
| 250m | 0.09 (0.07, 0.11)** | 250m | 0.10 (0.07, 0.128)** |
| 500m | 0.10 (0.07, 0.12)** | 500m | 0.08 (0.043, 0.11)** |
| 1000m | 0.15 (0.12, 0.18)** | 1000m | 0.14 (0.097, 0.174)** |
| 2000m | 0.30 (0.26, 0.34)** | 2000m | 0.24 (0.191, 0.285)** |

(B)

| English |  | Math |  |
| --- | --- | --- | --- |
| NDVI Buffer | Coefficient | NDVI Buffer | Coefficient |
| 250m | 0.28 (0.12, 0.44)** | 250m | 0.39 (0.19, 0.58)** |
| 500m | 0.49 (0.29, 0.70)** | 500m | 0.77 (0.52, 1.03)** |
| 1000m | 0.74 (0.52, 0.96)** | 1000m | 1.12 (0.85, 1.40)** |
| 2000m | 1.07 (0.83, 1.31)** | 2000m | 1.43 (1.13, 1.73)** |

**Table D. Coefficients (estimates with 95% confidence interval) of NDVI of (A) March, (B) July, and (C) October in GLMMs by using the CPI scores as the depended variable.** The coefficients are adjusted for race (percentage of different populations at a school, including African American, Asian, Hispanic, White, Native American, Native Hawaiian, and Non-Hispanic), gender (percentage of female student), language ability (percentage of first language not English), income level (percentage of low income student), student/teacher ratio, attendance, and location (county of school) in generalized linear mixed models. ** indicates P-value < 0.01.

(A)

| March |  |  |  |
| --- | --- | --- | --- |
| English (CPI scores) |  | Math (CPI scores) |  |
| NDVI Buffer | Coefficient | NDVI Buffer | Coefficient |
| 250m | 0.12 (0.10, 0.13)** | 250m | 0.08 (0.06, 0.10)** |
| 500m | 0.19 (0.17, 0.21)** | 500m | 0.08 (0.05, 0.11)** |
| 1000m | 0.22 (0.20, 0.25)** | 1000m | 0.10 (0.07, 0.13)** |
| 2000m | 0.24 (0.21, 0.26)** | 2000m | 0.10 (0.07, 0.13)** |

(B)

| July |  |  |  |
| --- | --- | --- | --- |
| English (CPI scores) |  | Math (CPI scores) |  |
| NDVI Buffer | Coefficient | NDVI Buffer | Coefficient |
| 250m | 0.05 (0.03, 0.06)** | 250m | 0.09 (0.07, 0.10)** |
| 500m | 0.02 (0.01, 0.03)** | 500m | 0.05 (0.03, 0.07)** |
| 1000m | 0.03 (0.02, 0.05)** | 1000m | 0.05 (0.03, 0.08)** |
| 2000m | 0.04 (0.02, 0.06)** | 2000m | 0.02 (-0.01, 0.05) |

(C)

| October |  |  |  |
| --- | --- | --- | --- |
| English (CPI scores) |  | Math (CPI scores) |  |
| NDVI Buffer | Coefficient | NDVI Buffer | Coefficient |
| 250m | 0 (-0.01, 0.01) | 250m | 0.02 (0.01, 0.04)** |
| 500m | -0.06 (-0.07, -0.04)** | 500m | 0.01 (-0.01, 0.03) |
| 1000m | -0.11 (-0.12, -0.09)** | 1000m | -0.01 (-0.03, 0.02) |
| 2000m | -0.15 (-0.17, -0.13)** | 2000m | -0.03 (-0.01, 0) |

**Table E. Earnings by educational attainment of USA.** Data are for persons age 25 and over. Earnings are for full-time wage and salary workers.

| Education attained | Median weekly earnings |
| --- | --- |
| Doctoral degree | $1,623 |
| Professional degree | 1,714 |
| Master's degree | 1,329 |
| Bachelor's degree | 1,108 |
| Associate's degree | 777 |
| Some college, no degree | 727 |
| High school diploma | 651 |
| Less than a high school diploma | 472 |
